# *Trichoderma* spp. promotes ginseng biomass by influencing the soil microbial community

**DOI:** 10.3389/fmicb.2024.1283492

**Published:** 2024-01-31

**Authors:** Linlin Zhang, Qiao Jin, Yiming Guan, Zhengbo Liu, Xiaoxi Pan, Yue Zhang, Yayu Zhang, Qiuxia Wang

**Affiliations:** ^1^Institute of Special Wild Economic Animal and Plant Sciences, Chinese Academy of Agricultural Sciences, Changchun, China; ^2^Jilin Provincial Key Laboratory of Traditional Chinese Medicinal Materials Cultivation and Propagation, Changchun, China; ^3^College of Pharmacy and Biological Engineering, Chengdu University, Chengdu, China

**Keywords:** ginseng, *Trichoderma* spp., biomass promotion, soil nutrients, rhizosphere microbiome

## Abstract

**Introduction:**

Ginseng (*Panax ginseng* C.A. Meyer) has multiple effects on human health; however, soil degradation seriously affects its yield. *Trichoderma* spp. play an important role in improving plant biomass by influencing the soil environment. Therefore, it is necessary to screen efficient *Trichoderma* strains that can increase ginseng biomass and determine their mechanisms.

**Methods:**

Herein, we selected six *Trichoderma* species (*T. brevicompactum*, *T. velutinum*, *T. viridescens*, *T. atroviride*, *T. koningiopsis*, and *T. saturnisporum*) isolated from ginseng rhizosphere soil, and evaluated their growth promoting effects on ginseng and their influence on the microbiome and chemical attributes of the ginseng rhizosphere soil.

**Results:**

Except for *T. saturnisporum* (F), compared with the control, the other five species increased ginseng biomass. In terms of chemical properties, the pH value, available potassium content, and available phosphorus content in the ginseng rhizosphere soil increased by 1.16–5.85%, 0.16–14.03%, and 3.92–38.64%, respectively, after root irrigation with spores of *Trichoderma* species. For the soil microbiome, fungal Chao1 and Ace richness indices decreased. Application of *Trichoderma* enhanced the relative level of *Proteobacteria*, but reduced the relative level of *Ascomycota*. At the genus level, application of *Trichoderma* enhanced the relative levels of *Sphingomonas, Blastomonas*, and *Trichoderma*, but reduced the relative level of *Fusarium*. Available K and available P were the most important elements that affected the structure of the bacterial community, while total K was the most influential element for the structure of the fungal community structure.

**Conclusion:**

The results indicated that the application of *Trichoderma* spp. could increase soil nutrients and regulate the structure and composition of the soil microbial community, thereby enhancing the biomass of ginseng. The results will provide guidance for soil improvement in ginseng cultivation.

## 1 Introduction

Ginseng has multiple beneficial effects on human health (Yu et al., 2017; Riaz et al., 2019). Ginseng has been artificially cultivated in large areas because of its high medicinal value (Sun et al., 2017). However, over time, cultivation of ginseng has led to decreased soil nutrients and beneficial soil microorganisms, but an increase in harmful microorganisms, especially those causing soil-borne diseases (Wang et al., 2020). To reduce the incidence of diseases and increase production, chemical fertilizers and pesticides have been widely used; however, this has caused problems such as persistence of pesticide residues and environmental pollution (Tang, 2020; Kai and Adhikari, 2021). Therefore, how to promote ginseng growth safely and effectively has become an important issue in ginseng cultivation (Chen et al., 2016).

*Trichoderma* spp. are widely distributed in soil, with functions such as inhibiting soil borne pathogens, improving soil, and promoting plant growth (Javeria et al., 2020; Jamil, 2021). Wu et al. (2022) found that *Trichoderma* applied after fumigation significantly improved cucumber yield and the soil chemical properties. Liu L. et al. (2022) found that biofertilizer containing *T. harzianum* increased the yield and quality of *Bupleurum chinense*, increased the content of available nutrients in the rhizosphere soil, and enhanced the activities of sucrase and catalase. *Trichoderma* spp. isolated from ginseng rhizosphere soil could improve the soil nutrient status and further affect the diversity of the soil fungal community (Ma et al., 2023).

However, there are few reports on whether *Trichoderma* species have broad spectrum biomass promoting effects on ginseng, and their relationship with the microbiome and physicochemical properties of ginseng rhizosphere soil. In the present study, we selected six *Trichoderma* species isolated from ginseng rhizosphere soil to evaluate their biomass promoting effects on ginseng and their influence on the microbiome and physicochemical properties of the ginseng rhizosphere soil. The results will provide guidance to improve the soil for ginseng cultivation and the development of biological fertilizers.

## 2 Materials and methods

### 2.1 Experimental materials

The *Trichoderma* strains were provided by the Institute of Special Wild Economic Animal and Plant Sciences, Chinese Academy of Agricultural Sciences (Changchun, China), and were also isolated from ginseng roots collected in Wanliang Town, Fusong County, Changbai City, Jilin Province, China. After morphological and molecular biological analyses, they were identified as: *T. brevicompactum, T. velutinum, T. viridescens, T. atroviride, T. koningiopsis*, and *T. saturnisporum*. Among them, *T. brevicompactum, T. velutinum, T. viridescens, T. atroviride*, and *T. koningiopsis* have been preserved in the China General Microbiological Culture Collection Center (preservation numbers: CGMCC NO. 23213, CGMCC No. 23211, CGMCC No. 23212, CGMCC No. 23214, and CGMCC No. 23210, respectively) (Wang et al., 2022a,b, c, d, e).

The *Trichoderma* strains were inoculated onto 90 mm potato dextrose agar (PDA) plates and incubated at 25°C for 7 days. After the spores were fully grown on the plate, an appropriate amount of sterile water was added to wash them off gently, followed by dispersal of the spores into a 6 g⋅L^–1^ sodium carboxymethyl cellulose (CMC) solution to obtain a spore suspension at 1 × 10^7^ colony forming units (CFU)⋅mL^–1^.

### 2.2 Experimental design

The experiment was conducted at the Wild Economic Animals and Plants Institute of CAAS, Changchun, China (E125°24′53″, N43°46′19″). Farmland soil was added to a 20 cm diameter pot, with 2 kg of soil per pot. The basic information of the foundation soils is shown in the FS treatment in Table 1. Same sized, healthy 1-year-old ginseng plants were selected for transplantation into the pots. The plants were grown in a room under a luminous intensity of 100 lux at 25°C and 60% humidity, and watered every 5 days. 7 treatments were set: A. *T. brevicompactum*, B. *T. velutinum*, C. *T. viridescens*, D. *T. atroviride*, E. *T. koningiopsis*, F. *T. saturnisporum* and CK [untreated plants (control)], respectively. Each treatment was performed using 4 replicates, with 5 plants per replicate. A total of 30 mL of prepared spore suspension of each fungal species was used for root irrigation after planting. Soil samples were collected after 90 days of ginseng seedling growth. We collected the rhizosphere soil and bulk soil in sterile plastic bags. The soil present within approximately 3 cm around the root of the ginseng seedling was considered the bulk soil, and the remaining soil attached to the ginseng roots was considered the rhizosphere soil. A portion of each soil sample was placed at −80°C for subsequent extraction of DNA, and the rest of each sample was dried naturally before analysis of its chemical attributes. The whole ginseng plant was washed thoroughly, and the fresh weight of the whole plant and roots were measured. The dry weight of the whole plant and roots were then measured after drying at 105°C.

**TABLE 1:** Effects of different treatments on soil nutrients (mean ± SE).

Soil properties	CK	A	B	C	D	E	F	FS
PH	5.13 ± 0.13b	5.37 ± 0.06ab	5.43 ± 0.05a	5.21 ± 0.07ab	5.26 ± 0.1ab	5.3 ± 0.07ab	5.19 ± 0.09ab	7.07 ± 0.07
TC (g/kg)	18.06 ± 0.02c	17.2 ± 0.05d	19.18 ± 0.11a	18.59 ± 0.23b	18.26 ± 0.16bc	16.37 ± 0.18e	17.29 ± 0.11d	19.36 ± 0.07
TN (g/kg)	0.82 ± 0.01b	0.48 ± 0.01e	0.92 ± 0.01a	0.75 ± 0.01c	0.73 ± 0.01c	0.59 ± 0.01d	0.75 ± 0.02c	0.66 ± 0.01
TP (g/kg)	2.11 ± 0.09a	2.03 ± 0.05a	2.18 ± 0.16a	2.0 ± 0.10a	2.13 ± 0.09a	1.96 ± 0.07a	2.06 ± 0.07a	2.01 ± 0.06
TK (g/kg)	5.21 ± 0.1ab	4.76 ± 0.15c	4.95 ± 0.05bc	4.97 ± 0.12bc	5.44 ± 0.11a	5.0 ± 0.07bc	5.04 ± 0.06bc	4.86 ± 0.04
NH4 + -N (mg/kg)	35.39 ± 0.57ab	30.43 ± 1.07c	31.32 ± 0.55bc	31.95 ± 1.84bc	37.33 ± 2.39a	32.68 ± 1.30bc	34.11 ± 0.59abc	31.33 ± 0.43
NO3–N (mg/kg)	62.42 ± 3.57bc	48.34 ± 8.29bc	62.05 ± 5.24bc	43.89 ± 12.56c	75.33 ± 6.69ab	53.69 ± 12.71bc	93.69 ± 10.61a	39.17 ± 10.21
AP (mg/kg)	202.53 ± 3.54b	214.4 ± 0.94ab	221.05 ± 4.89a	202.85 ± 6.69b	230.95 ± 5.68a	221.3 ± 4.28a	227.85 ± 9.51a	149.15 ± 9.04
AK (mg/kg)	177.55 ± 2.57d	200.43 ± 19.03bcd	192.47 ± 13.62cd	184.52 ± 11.86cd	227.27 ± 12.58abc	246.16 ± 2.5a	237.21 ± 22.29ab	139.98 ± 3.57
S (g/kg)	0.78 ± 0.3a	0.37 ± 0.04b	0.42 ± 0.03ab	0.29 ± 0.02b	0.48 ± 0.05ab	0.35 ± 0.05b	0.54 ± 0.06ab	0.69 ± 0.07

Different lowercase letters are statistically significant at *P* < 0.05.

### 2.3 Analysis of soil chemical properties

The soil pH value was measured using a pH/oxidation reduction potential (ORP) acidity meter. The ammonium nitrogen (NH_4_^+^-N), nitrate nitrogen (NO_3_^–^-N), total nitrogen (TN), and total carbon (TC) contents were determined as described previously (Jin et al., 2022a). The salt content (S) was determined using a conductivity meter, and the agricultural soil chemical analysis method (Bao, 2000) was used to determine the total potassium (TK), total phosphorus (TP), available potassium (AK), and available phosphorus (AP).

### 2.4 Soil DNA extraction, PCR, and sequencing

A MoBio Laboratories PowerSoil DNA Isolation Kit (MoBio Laboratories, Carlsbad, CA, USA) was employed to extract DNA. The 16S rRNA in the DNA sample was amplified using the primers 806R (GGACTACHVGGGTWTCTAAT) and 338F (ACTCCTACGGGAGGCAGCA) (Fu et al., 2023) using the following reaction conditions: 95°C for 10 min, followed by 40 cycles of 15 s at 95°C, 60 s at 55°C, and 90 s at 72°C, with a final extension of 7 min at 72°C. Likewise, primers ITS1F (CTTGGTCATTTAGAGGAAGTAA) and ITS2 (GCTGCGTTCTTCATCGATGC) (White et al., 1990; Gardes and Bruns, 1993) were used to amplify the internal transcribed spacer (ITS) region as follows: 95°C for 5 min, then 35 cycles of 95°C for 1 min, 53°C for 45 s, and 72°C for 1 min. The amplicons were purified and then subjected to Illumina MiSeq sequencing (Illumina Inc., San Diego, CA, USA).

First, the original data was filtered employing Trimmomatic 0.33 (Bolger et al., 2014), and primer sequences were removed employing Cutadapt 1.9.1 (Martin, 2011). Subsequently, Usearch (version 10) (Edgar, 2013) was employed to splice the double-ended reads, with chimeras being removed using UCHIME (version 4.2) (Edgar et al., 2011) to leave sequence of high quality for subsequent analysis. Using a cutoff of 97% similarity, the sequences were clustered into operational taxonomic units (OTUs) using Usearch. OTUs with counts less than two in all samples were filtered out. Taxonomy annotation of the resulting OTUs was carried out using the Naive Bayes classifier in QIIME2 (Bolyen et al., 2019), utilizing the SILVA database (release 138.1) (Quast et al., 2013), with a confidence threshold of 70%. Meanwhile, Alpha and Beta diversity analyses were performed using the QIIME2 software to assess the species diversity within each sample. This involved calculating the Shannon, Simpson, ACE, and Chao1 indices to obtain information on the diversity of species within the samples. Additionally, information on the common and unique OTUs between different samples or groups was obtained.

### 2.5 Statistical analysis

SAS version 9.1 (SAS institute, Cary, NC, USA) was employed to analyze the soil bacteria and fungi diversity indices (e.g., Shannon and Chao1) and soil chemical attributes. One-way analysis of variance (ANOVA) with the least significance difference (LSD) test were used to compare the mean vales for the samples and variability in the data was expressed as the standard error (*n* = 4). Differences at *P* < 0.05 or *P* < 0.01 were considered statistically significant. We performed linear discriminant analysis Effect Size (LEfSe) analysis according to the method of Segata et al. (2011). Redundancy analysis (RDA) of soil chemical factors and microbial diversity was carried out using CANOCO 5.0. The environmental variables were evaluated using a partial Monte Carlo permutation test (499 permutations) with an unrestricted permutation to investigate their statistical significance (Huang et al., 2017).

## 3 Results

### 3.1 Effects of different treatments on the fresh weight and dry weight of ginseng plants

**FIGURE 1:**
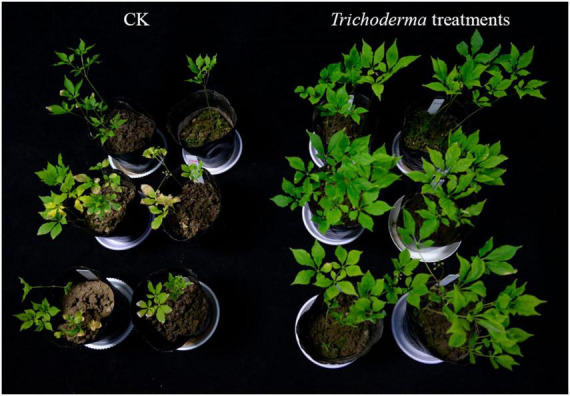
Characterization of ginseng plants under different treatments.

As shown in Figure 1, the control group showed symptoms of ginseng wilt disease, with dry leaf tips and edges, while the symptoms of ginseng wilt disease in *Trichoderma* treatment groups were almost non-existent or reduced. Compared with CK, the fresh and dry weight of ginseng under A and E treatments increased significantly (*P* < 0.05), while the dry weight of ginseng under B, C, and D treatments increased significantly (*P* < 0.05). The fresh weight of ginseng under A treatment increased by 20.73% and the dry weight increased by 49.02%. The fresh weight of ginseng under E treatment increased by 15.45% and the dry weight increased by 35.30%, the dry weight of ginseng under B, C, and D treatments increased by 45.10, 35.30, and 49.02%, respectively (Table 2). Thus, treatment with A had the best effect on increasing the fresh and dry weight of ginseng, followed by E treatment, and B, C, and D treatments were beneficial only for the dry weight accumulation of ginseng. There was no significant difference between F treatment and the control group.

**TABLE 2:** Effects of different treatments on fresh weight and dry weight (mean ± SE).

Treatment	Fresh weight (g)	Dry weight (g)
CK	2.46 ± 0.17cd	0.51 ± 0.07b
A	2.97 ± 0.13a	0.76 ± 0.12a
B	2.77 ± 0.29abc	0.74 ± 0.08a
C	2.71 ± 0.22abc	0.69 ± 0.09a
D	2.57 ± 0.30bcd	0.76 ± 0.06a
E	2.84 ± 0.09ab	0.69 ± 0.03a
F	2.36 ± 0.15d	0.51 ± 0.07b

CK, without treatment. A, *T. brevicompactum*; B, *T. velutinum*; C, *T. viridescens*; D, *T. atroviride*; E, *T. koningiopsis*; F, *T. saturnisporum.* Different lowercase letters are statistically significant at *P* < 0.05.

### 3.2 Comparisons of soil chemical properties among different treatments

The soil chemical properties of 1-year-old ginseng treated with different *Trichoderma* spp. after a 90-day growth period are shown in Table 1. The pH value of the soil increased by 1.16–5.85% compared with CK. The TC under B, C, and D treatments was increased compared with that in CK; the TN under B treatment increased significantly compared with that of CK (*P* < 0.05); the TP under B and D treatments increased compared with that of CK; and the contents of TK and NH_4_^+^-N under D treatment increased compared with that of CK. The NO_3_^–^-N content under D and F treatments increased compared with that of CK. The contents of AP and AK under all treatments increased by 0.16–14.03% and 3.9–38.64% compared with CK, respectively. The S content under each treatment was decreased compared with that of CK, demonstrating that *Trichoderma* application reduced the S content, in which treatments A, C, and E resulted in significant differences compared with CK (*P* < 0.05).

### 3.3 Diversity analysis of bacteria and fungi in the soil after various treatments

The indexes of diversity coverage of microorganisms in the soil were all > 0.97, showing that the results of sequencing accurately represented the actual situation of the soil bacteria and fungi. For bacteria, except for treatment C, the Shannon index was higher in all treatments compared with that of CK. The Ace indices of treatments A, B, and D were higher than that of CK. The Chao1 indices of treatments A and B were higher than that of CK. For fungi, except for treatment E, the Shannon index was lower than that in CK for all treatments, while the Simpson indices of treatments D and E were higher than that of CK. The Ace and Chao1 indices of all treatments were lower than that of CK (*P* < 0.05). Therefore, overall, the application of *Trichoderma* increased bacterial diversity and reduced fungal diversity (Table 3).

**TABLE 3:** Soil bacterial and fungal diversity indices under different treatments.

Treatment	Shannon index		Simpson index		Ace index		Chao1 index	
	**Bacteria**	**Fungi**	**Bacteria**	**Fungi**	**Bacteria**	**Fungi**	**Bacteria**	**Fungi**
CK	8.95a	6.58a	0.99a	0.96a	1810.99a	762.41a	1826.96a	790.73a
A	9.09a	5.57b	0.99a	0.93a	1838.67a	523.12c	1853.89a	543.55b
B	8.98a	6.13ab	0.99a	0.95a	1820.96a	557.90bc	1841.39a	545.74b
C	8.92a	6.44ab	0.99a	0.96a	1799.02a	655.25ab	1813.99ab	650.47b
D	9.09a	6.54a	0.99a	0.97a	1814.13a	680.49a	1824.90a	661.53b
E	8.97a	6.83a	0.99a	0.98a	1735.48b	538.69bc	1753.89b	539.98b
F	9.07a	6.55a	0.99a	0.96a	1777.76ab	541.55bc	1789.19ab	532.92b

Different lowercase letters are statistically significant at *P* < 0.05.

### 3.4 Soil microbial community composition among different treatments

#### 3.4.1 Phylum level classification of bacteria and fungi

**FIGURE 2:**
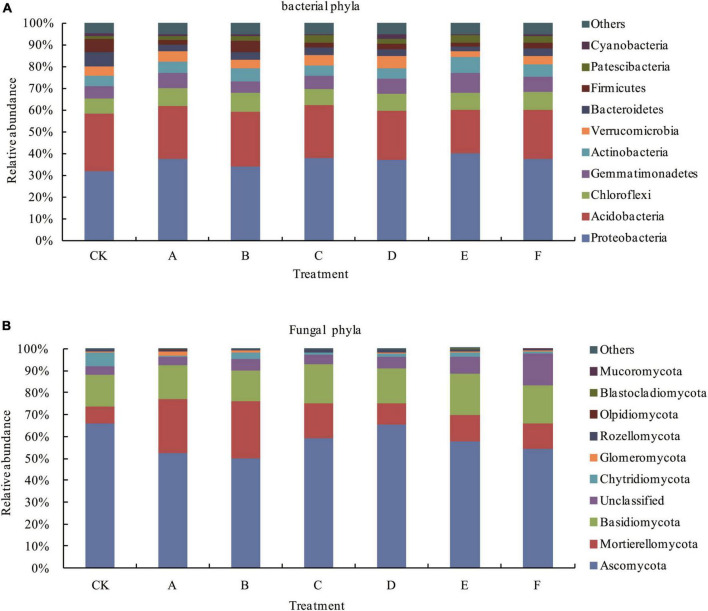
Comparative levels of bacterial **(A)** and fungal **(B)** phyla. Others: all species other than the top 10 species according to their relative abundance levels.

After applying *Trichoderma*, there was no change in the top 10 bacterial population categories compared with those in CK; however, there were certain changes in their relative abundances, and the changes varied with different *Trichoderma* treatments. The top 10 soil bacterial phyla by relative abundance under the various treatments were: *Proteobacteria*, *Acidobacteria*, *Chloroflexi*, *Gemmatimonadetes*, *Actinobacteria*, *Verrucomicrobia*, *Bacteroidetes*, *Firmicutes*, *Patescibacteria*, and *Cyanobacteria*. The sum of the comparative levels of *Acidobacteria* and *Proteobacteria* reached 58.48–62.45% (Figure 2A). The comparative levels of *Chloroflexi* and *Proteobacteria* in each *Trichoderma* treatment group were increased compared with those in CK. Among them, the relative abundance of *Proteobacteria* under E treatment was the highest, and the relative abundance of *Chloroflexi* under B treatment was the highest; however, the relative abundance of *Acidobacteria* under all treatments was decreased compared with that in CK. Except for treatment B, the relative abundance of *Gemmatimonadetes* under all treatments was increased compared with that CK.

After applying *Trichoderma*, there was no change in the top 10 fungal population categories compared with that in CK; however, there were certain changes in their relative abundances, and the changes varied with different *Trichoderma* treatments. The top 10 soil fungal phyla by relative abundance under the various treatments were: *Ascomycota*, *Mortierellomycota*, *Basidiomycota*, Unclassified, *Chytridiomycota*, *Glomeromycota*, *Rozellomycota*, *Olpidiomycota*, *Blastocladiomycota*, and *Mucoromycota*. The sum of the comparative levels of *Ascomycota*, *Mortierellomycota*, and *Basidiomycota* reached 83.52–93.01% (Figure 2B). The relative abundance of *Ascomycota* in each *Trichoderma* treatment group was lower than that in CK and the relative abundance of *Mortierellomycota* was higher than that in CK. Except for treatment B, the relative abundance of *Basidiomycota* under all treatments was increased compared with that in CK.

#### 3.4.2 Genus level classification of bacteria and fungi

**FIGURE 3:**
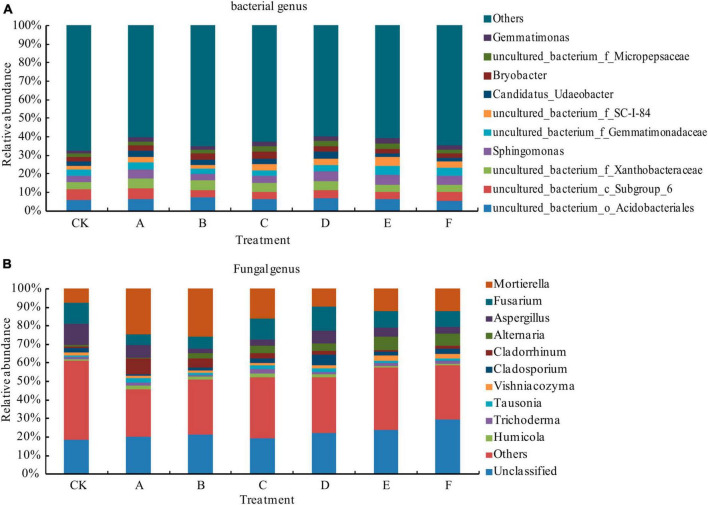
Comparative levels of bacterial **(A)** and fungal **(B)** genera. Others: all species other than the top 10 species according to their relative abundance levels.

As shown in Figure 3A, the bacterial community comprised a large number of rare species (Other) and Uncultured bacteria, with relatively low relative abundance and rich species. Except for treatment B, the relative abundance of *Sphingomonas* under *Trichoderma* treatment was increased compared with that in CK. The relative abundance of *Gemmatimonas* under each treatment was increased compared with that in CK, and except for treatments E and F, the comparative level of *Bryobacter* was increased compared with that in CK.

As shown in Figure 3B, for fungal genera, the comparative level of Unclassified and Other fungi were high, indicating that there were more unclassified flora and rare species under each treatment, and the sum of their relative abundances reached 45.8–61.3%. The comparative level of *Mortierella* under each *Trichoderma* treatment was higher than that in CK. Except for treatment D, the comparative level of *Fusarium* was lower under *Trichoderma* treatment than that in CK, and except under treatment D, the comparative level of *Trichoderma* was increased compared with that in CK.

### 3.5 Difference analysis of the microbial communities in the soil

**FIGURE 4:**
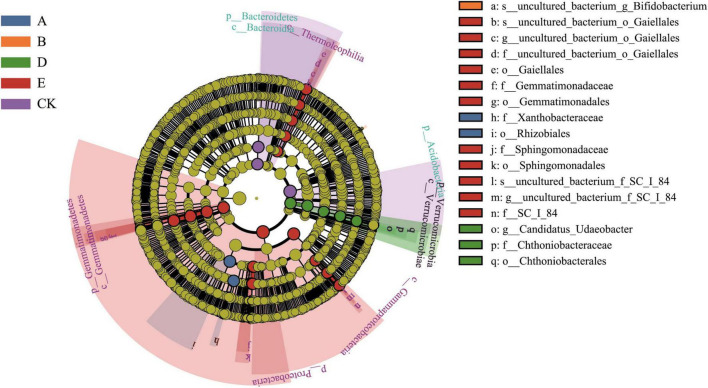
LEfSe analysis of the structure of the soil sample bacterial communities. The circle, from inside to outside, indicates bacteria from phylum to species, respectively. Yellow points indicate that bacteria had no notable differences among the treatment groups, and biomarker bacteria in the different treatments were classified using different colors.

**FIGURE 5:**
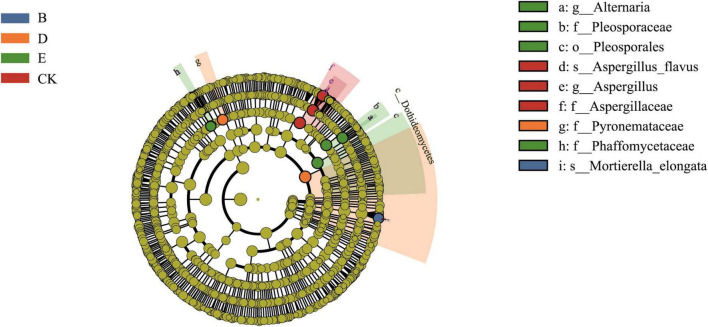
LEfSe analysis of the fungal community in soil samples. The circle, from inside to outside, indicates fungi from phylum to species, respectively. Yellow points indicate that fungi had no notable differences among the treatment groups, and biomarker fungi in the different treatments were classified using different colors.

The LEfSe statistical result analysis (Figure 4) indicated that the bacterial species under C and F treatments did not differ significantly, thus these two groups were omitted from the analysis. *Xanthobacteraceae* and *Rhizobiales* in group A; *Bifidobacterium* in group B; *Udaeobacter* and *Chthoniobacterales* in group D; *Proteobacteria*, *Gemmatimonadaceae*, *Sphingomonadaceae*, and *Thermoleophilia* in Group E; and *Acidobacteria* and *Bacteroidia* in Group CK were identified as biomarkers. The fungal species under A, C, and F treatments did not differ significantly, thus these groups were omitted from the analysis. *Mortierella* in group B, *Pyronemataceae* in group D, *Alternaria* and *Pleosporaceae* in group E, and *Aspergillus* in group CK were identified as biomarkers (Figure 5).

### 3.6 RDA of the microbial communities and nutrients in the soil under different treatments

**FIGURE 6:**
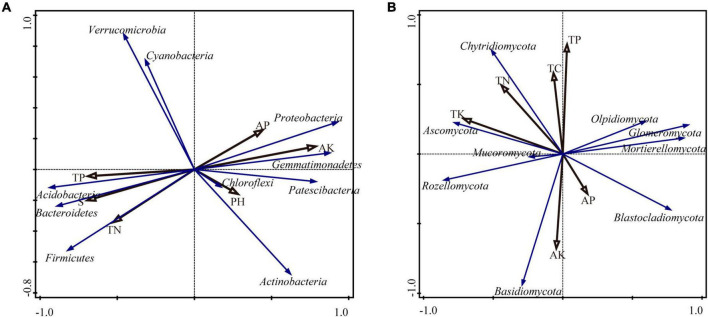
Analysis of redundancy for soil nutrients and the soil bacterial **(A)** and fungal **(B)** communities.

Figure 6A shows the RDA of the soil bacterial community structure and soil chemical properties. The first two axes of the RDA plot explain 53.31 and 22.09% of the total variance, respectively (75.40% combined). The Monte Carlo test results showed that available potassium (*F* = 2.7, *P* = 0.018) was the most important factor affecting the soil bacterial community, and 95% of the bacterial community variation between samples could be explained by this environmental factor. The environmental factors exerted their effects, from large to small, in the order: available potassium, salt, available phosphorus, pH value, and total nitrogen. As shown in Figure 6A, available potassium and available phosphorus correlated positively with the relative abundance of *Proteobacteria* and *Gemmatimonadetes*, and negatively with the relative abundance of *Firmicutes*.

Figure 6B shows the RDA analysis of the soil structure of the fungal community structure and chemical properties of the soil. The first two axes of the RDA plot explain 47.29 and 20.00% of the total variance, respectively (67.29% combined). The Monte Carlo test results showed that total potassium (*F* = 2.3, *P* = 0.064) and total nitrogen (*F* = 3.5, *P* = 0.062) were the most important factors affecting the soil fungal community, and 98.3% of the fungal community variation between samples could be explained by these environmental factors. The environmental factors exerted their effects, from large to small, in the order: total potassium, total nitrogen, total phosphorus, available phosphorus, and available potassium. As shown in Figure 6B, total potassium and total nitrogen were positively correlated with *Ascomycota* and *Chytridiomycota* and negatively correlated with *Blastocladiomycota.*

## 4 Discussion

Soil degradation seriously affects the yield and quality of ginseng. *Trichoderma* spp. play an important role in improving plant biomass by influencing the soil environment (Meng et al., 2019; Wang et al., 2021). Therefore, it is necessary to screen high-efficiency *Trichoderma* strains that can increase the biomass of ginseng. Previous research demonstrated that *T. koningiopsis* could promote plant growth via increased levels of volatile organic compounds (You et al., 2022). After treatment with *T. atroviride*, the aerial and root dry weights of tomato increased (Rao et al., 2022). Herein, we found that five out of six *Trichoderma* species isolated from ginseng rhizosphere soil showed biomass promoting effects on ginseng plants, among which *T. brevicompactum* had the best effect on increasing ginseng fresh and dry weight, followed by *T. velutinum* and *T. koningiopsis*; and *T. atroviride* was beneficial to dry weight accumulation of ginseng. These results identified excellent *Trichoderma* materials to improve ginseng production.

Microorganisms are an important component of soil, and are closely related to soil health and quality (de Vries et al., 2020). Studies have shown that *Trichoderma* spp. can alter the soil microbiome. Zhang et al. (2020) found that inoculation with *T. asperellum* reduced fungal diversity and increased bacterial diversity. In particular, it increased the relative abundance of rhizosphere microorganisms that promote plant growth, such as *Sphingomonas*,*Trichoderma*,*Actinomadura*, *Pseudomonas*, and *Rhodanobacter*. Li et al. (2023) found that dual inoculation with dark septate endophytes and *T. koningiopsis* altered the microbial community structure in the rhizosphere, in which the levels of *Acidobacteriae*, *Ascomycota*, *Firmicutes*, and *Actinobacteriota* increased significantly, resulting in *Vicinamibacteria* and *Trichoderma* being enriched in the soil. After fumigation, *Trichoderma* application enhanced the relative abundance of beneficial microorganisms, which can improve the soil microbiome (Wu et al., 2022). In this study, we found that *Trichoderma* application reduced fungal richness, which might have been caused by the antagonism of *Trichoderma* against some pathogenic fungi. At the phylum level, all six *Trichoderma* species enhanced *Proteobacteria* levels, which were highest under treatment with *T. koningiopsis* (E). Certain *Proteobacteria* have major functions in increasing plant yield, such as nitrogen fixation, phosphorus solubilization, and plant growth promotion (Huang et al., 2020; Martínez, 2023), which help ginseng to absorb and utilize nutrients in the soil. At the genus level, all six *Trichoderma* species increased the relative abundance of *Gemmatimonas*. Except for treatment B, they increased the relative abundance of *Sphingomonas* and *Trichoderma*, and except for treatment D, they decreased the relative abundance of *Fusarium*. There was a significant and positive correlation between *Gemmatimonas* and soil nutrient components, and thus *Gemmatimonas* might be an indicator genus in response to changes in soil nutrient contents. Besides, *Gemmatimonas* can solubilize insoluble elements, such as phosphorus, and induce plant stress resistance or produce antifungal antibiotics, which have been proven to promote plant growth and enhance nutrient uptake (Shang and Liu, 2020; Liu C. et al., 2022). Recent studies have detailed the role of *Sphingomonas* species in plant growth promotion via the production of phytohormones and increased stress tolerance (Matsumoto et al., 2021; Jin et al., 2022b; Lombardino et al., 2022). Meanwhile, the relative abundance of *Trichoderma* increased after inoculation, indicating that *Trichoderma* could grow and reproduce in the treated soil. Root rot is the main disease responsible for decreases in the yield and quality of ginseng, and the main pathogen causing root rot is *Fusarium* (Guan et al., 2014; Wang et al., 2016). *Fusarium* is a common plant pathogen that can hinder the growth of many crops (Pegg et al., 2019; Srinivas et al., 2019; Kong et al., 2023). The observed decrease of*Fusarium* in the soil showed that the addition of *Trichoderma* inhibited *Fusarium* and could prevent root rot of ginseng. Therefore, the results of our study, combined with those of previous studies, indicated that inoculation with *Trichoderma* could promote the growth of beneficial microorganisms and decrease the proliferation of deleterious microbes.

Soil pH value is a key indicator of ginseng planting site selection (Kim et al., 2015), and many soil borne diseases are affected by the soil pH value. A decreased in soil pH might increase the incidence of ginseng root diseases (Jin et al., 2022a). Herein, we demonstrated that *Trichoderma* inoculation increased soil PH value, and the available phosphorus and available potassium contents, which was conducive to the prevention and control of ginseng soil-borne diseases and the promotion of ginseng growth. Thus, inoculation with *Trichoderma*, which colonized and grew in the soil and would secrete organic acids and other factors, changed the pH and increased the available phosphorus and available potassium content (Tekaya et al., 2018). In this study, *Trichoderma* species had different effects on soil physicochemical properties, among which *T. velutinum* (B), *T. atroviride* (D), and *T. koningiopsis* (E) improved the physicochemical properties. Parada et al. (2019) showed that inoculating microbials into acidic soil could alleviate soil acidification, which agreed with our findings. The enhancement of available phosphorus and available potassium contents might also have been caused by the activation of beneficial microbial populations in the soil after inoculation with *Trichoderma* (Qi et al., 2022). Beneficial microorganisms and their activities facilitate the transformation of plant nutrients from non-effective forms to effective forms, thereby improving soil fertility (Jain et al., 2015; Yuan et al., 2016). Saravanakumar et al. (2013) found that *Trichoderma* has a solubilizing effect on phosphate. Mao and Jiang (2021) reported that the contents of alkali-hydrolysable nitrogen, organic matter, available potassium, and available phosphorus in soil increased after application of *T. hamatum*, which was consistent with our results.

Soil properties might influence the structure and diversity of soil microbial communities (Khan et al., 2016; Sun et al., 2019). In this study, RDA was carried out at the species level for bacteria and fungi, and among soil nutrients, available K and available P were the most influential elements for the structure of the bacterial community, whereas total K had the largest influence the structure of the fungal community. This was consistent with the results of Zhu and Yu (2022). Available potassium and available phosphorus correlated positively with *Proteobacteria* and *Blastomonas* levels, indicating that application of *Trichoderma* could increase the contents of available potassium and phosphorus in ginseng soil, and increase the comparative levels of *Proteobacteria* and *Blastomonas* in soil, thus promoting ginseng growth.

## 5 Conclusion

The results of this study indicated that different *Trichoderma* spp. have different effects on the biomass of ginseng, and the chemical properties and microbiome of ginseng soil. Most of the selected *Trichoderma* spp. had beneficial effects on ginseng biomass, improved soil nutrients, increased the relative abundance of beneficial microbial populations (such as *Gemmatimonas*, *Sphingomonas*, and *Trichoderma*), and reduced the relative abundance of harmful microbial populations (such as *Fusarium*). In particular, *T. koningiopsis* (E) was superior to the other tested species and has potential for application. This study provides a theoretical basis for soil improvement and biological control of diseases in ginseng cultivation. However, the limitations of greenhouse cultivation mean that additional long-term field experiments should be conducted with different climatic conditions.

## Data availability statement

The datasets presented in this study can be found in online repositories. The names of the repository/repositories and accession number(s) can be found below: https://www.ncbi.nlm.nih.gov/, PRJNA1064685, PRJNA1064678.

## Author contributions

LZ: Data curation, Methodology, Validation, Visualization, Writing—original draft. QJ: Data curation, Formal analysis, Software, Writing—review and editing. YG: Conceptualization, Data curation, Formal analysis, Writing—review and editing. ZL: Data curation, Formal analysis, Methodology, Writing—review and editing. XP: Resources, Validation, Writing—review and editing. YuZ: Data curation, Methodology, Software, Writing—review and editing. YaZ: Data curation, Methodology, Validation, Writing—review and editing. QW: Conceptualization, Formal analysis, Funding acquisition, Methodology, Project administration, Resources, Supervision, Validation, Visualization, Writing—review and editing.
